# Identification of a MicroRNA Signature Associated With Lymph Node Metastasis in Endometrial Endometrioid Cancer

**DOI:** 10.3389/fgene.2021.650102

**Published:** 2021-04-15

**Authors:** Kaiyou Fu, Yanrui Li, Jianyuan Song, Wangyu Cai, Wei Wu, Xiaohang Ye, Jian Xu

**Affiliations:** ^1^School of Medicine, Zhejiang University, Hangzhou, China; ^2^School of Control Science and Engineering, Zhejiang University, Hangzhou, China; ^3^Fourth Affiliated Hospital, School of Medicine, Zhejiang University, Hangzhou, China; ^4^Women’s hospital, School of Medicine, Zhejiang University, Hangzhou, China

**Keywords:** endometrial cancer, miRNA expression profile, lymph node metastasis, molecular biomarker, TCGA

## Abstract

**Background:**

Lymph node metastasis (LNM) is an important prognostic factor in endometrial cancer. Anomalous microRNAs (miRNAs) are associated with cell functions and are becoming a powerful tool to characterize malignant transformation and metastasis. The aim of this study was to construct a miRNA signature to predict LNM in endometrial endometrioid carcinoma (EEC).

**Method:**

Candidate target miRNAs related to LNM in EEC were screened by three methods including differentially expressed miRNAs (DEmiRs), weighted gene co-expression network analysis (WGCNA), and decision tree algorithms. Samples were randomly divided into the training and validation cohorts. A miRNA signature was built using a logistic regression model and was evaluated by the area under the curve (AUC) of receiver operating characteristic curve (ROC) and decision curve analysis (DCA). We also conducted pathway enrichment analysis and miRNA–gene regulatory network to look for potential genes and pathways engaged in LNM progression. Survival analysis was performed, and the miRNAs were tested whether they expressed differently in another independent GEO database.

**Result:**

Thirty-one candidate miRNAs were screened and a final 15-miRNA signature was constructed by logistic regression. The model showed good calibration in the training and validation cohorts, with AUC of 0.824 (95% CI, 0.739–0.912) and 0.821 (95% CI, 0.691–0.925), respectively. The DCA demonstrated the miRNA signature was clinically useful. Hub miRNAs in signature seemed to contribute to EEC progression *via* mitotic cell cycle, cellular protein modification process, and molecular function. MiR-34c was statistically significant in survival that a higher expression of miR-34c indicated a higher survival time. MiR-34c-3p, miR-34c-5p, and miR-34b-5p were expressed differentially in GSE75968.

**Conclusion:**

The miRNA signature could work as a noninvasive method to detect LNM in EEC with a high prediction accuracy. In addition, miR-34c cluster may be a key biomarker referring LNM in endometrial cancer.

## Introduction

Endometrial cancer is the fourth most often diagnosed malignancy in the female population worldwide. Estimated numbers of new cases and deaths in 2020 in the United States were 65,620 and 12,590, respectively (Siegel et al., 2020). Endometrial endometrioid carcinoma (EEC) is the most common histological type of endometrial cancer (Creasman et al., 2006). Lymph node metastasis (LNM) is a key determinant of the prognosis and treatment of EEC. It was reported that 5-year survival of patients whose tumor was limited in the uterine corpus was 80–90%, while those with LNM was 50–60% (Creasman et al., 2006; Lewin and Wright, 2011). Therefore, lymph node evaluation is critical for diagnosis and further adjuvant therapy. Lymphadenectomy used to be the routine therapy for EEC and was critical for surgical staging. However, evidence shows that lymphadenectomy may be unnecessary for early-stage EEC because of limited benefits and may lead to nerve injury, prolonged operation time, lymphedema, blood loss, and lymph cyst formation (Morrow et al., 1991; Homesley et al., 1992; Orr et al., 1997; Abu-Rustum et al., 2006). Therefore, a more selective lymphadenectomy is applied, and new noninvasive ways to evaluate lymph node status before surgery need to be explored.

MicroRNAs (miRNAs) are small RNA molecules that posttranscriptionally regulate gene expression by guiding target mRNA cleavage or translational inhibition. Multiple studies have shown that miRNAs play significant roles in the occurrence, development, and prognosis of cancer, making them potential markers for diagnosing specific cancers and progression (Cai et al., 2009; Chan et al., 2011). For example, a miRNA signature consisting of miR-155, miR-21, and 33 other miRNAs was found to distinguish clear-cell kidney cancer from normal kidney tissue with high confidence (Juan et al., 2010). The specific miRNA panels also have good performance on the prediction of prognosis of colon cancer, liver cancer, and lung cancer (Budhu et al., 2008; Hur et al., 2015; Cen et al., 2020). Previous studies have tried to determine the miRNAs associated with EEC compared to normal endometrial tissue (Tsukamoto et al., 2014; Wang Q. et al., 2020). However, few studies have worked on LNM evaluation in EEC using miRNA signatures. Therefore, the aim of this study was to evaluate whether miRNA profiles can predict LNM and to identify candidate target miRNAs and their relations to LNM progression in EEC.

## Materials and Methods

### Study Workflow

**FIGURE 1:**
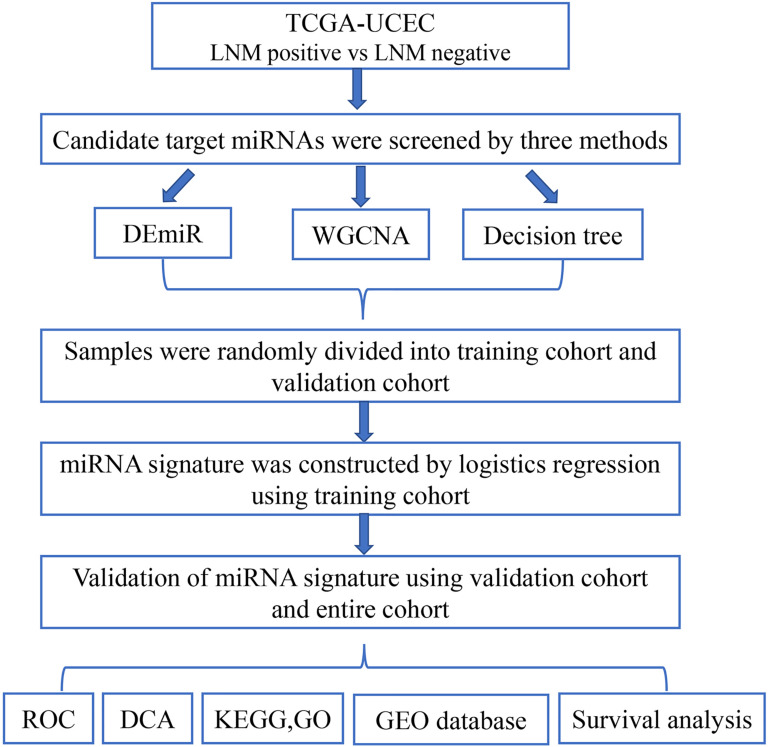
Flowchart of the study. This figure describes the flow sheet of the study. LNM, lymph node metastasis; DEmiR, differentially expressed microRNA; WGCNA, weighted gene co-expression network analysis; ROC, receiver operating characteristic curve; DCA, decision curve analysis; GO, Gene Ontology; KEGG, Kyoto Encyclopedia of Genes and Genomes.

The schematic of study workflow was shown in Figure 1. Clinical data and miRNA profile were obtained from The Cancer Genome Atlas (TCGA). Three methods including differentially expressed miRNAs (DEmiRs), weighted gene co-expression network analysis (WGCNA), and decision tree algorithms were performed between LNM-positive group and LNM-negative group to screen candidate target miRNAs. Samples from TCGA were randomly divided into training and validation cohorts. A miRNA signature was built using logistic regression model in the training cohort. The performance of the miRNA signature was evaluated by receiver operating characteristic curve (ROC) and decision curve analysis (DCA). Pathway enrichment analysis and miRNA–gene regulatory network were constructed to look for potential genes and pathways engaged in LNM progression. The expression of miRNAs in signature was validated in another independent Gene Expression Omnibus (GEO) database. Finally, survival analysis was performed to explore the prognosis meaning of the identified miRNAs.

### The Cancer Genome Atlas miRNA Expression Profiles

Transcriptome data including miRNA expression and mRNA expression for EEC were obtained from TCGA (TCGA-UCEC) for count data type^1^. The corresponding clinical data, including age, stage, and histological type and grade, were also collected. Only cases involving a histologic EEC diagnosis and with complete clinical information regarding tumor grade and lymph node status were selected for analysis. Additionally, we selected only patients with clinical stage I (negative lymph nodes) or IIIC (positive lymph nodes) disease for comparison.

### Screening Candidate miRNA

Three methods were used to screen candidate miRNA related to LNM in EEC including DEmiR, WGCNA, and decision tree algorithms, which were combined to come up with a union set of candidate miRNAs for further analysis.

#### Differential Expression Analysis

The downloaded data of miRNAs were standardized, and then edge R package was used for differential expression analysis. The screening criteria were |fold change|>2 and false discovery rate (FDR) < 0.05.

#### Construction of Co-expression Network

Weighted gene co-expression network analysis was aimed to form the modules of co-expression gene for the EEC-related networks and interactions (Langfelder and Horvath, 2008). Following the protocols of WGCNA, the networks were constructed based on the weighted correlation matrices. Briefly, the gene expression profiles were transformed into connection weights that can be visualized as topology overlap measures (TOMs). We selected the module most relevant to LNM and then screened target miRNA in the chosen module.

#### Decision Tree Algorithms

Decision tree algorithms are widely used for detecting the important features in classification in the machine learning field (Monteiro and Murphy, 2011). In our research, we applied decision tree algorithms to identify target miRNA related to LNM. Light-GBM, a state-of-the-art Gradient Boosting Decision Tree (GBDT) algorithm, was used as our feature-ranking algorithm (Ke et al., 2017). Features were ranked according to the feature importance value, which is defined as the number of times a feature is selected as a partition point. To ensure that the final ranking of features is reliable, the process was repeated 1,000 times. In each cycle, learning rate, feature fraction, and bagging fraction were set randomly between 0.005 and 0.015, 0.7 and 1, and 0.7 and 1, respectively.

### Model Construction and Validation

Patients in TCGA-UCEC dataset were randomly divided into training and validation cohorts, with *t* test and chi-square test proving no significant difference of patients’ characteristics between the two cohorts. Logistic regression analysis was used in the training cohort to form the miRNA signature. After removing miRNAs that contributed little to the prediction of LNM, the final miRNA signature was defined. Then, the logistic regression formula was applied to the validation cohort, and a risk score of LNM was calculated. ROC was constructed, and the area under the curve (AUC) was calculated to validate the performance of prediction. DCA was conducted by R studio in order to evaluate the clinical application value of the signature.

### The Gene Ontology Annotation and Kyoto Encyclopedia of Genes and Genomes Analysis of miRNAs in the Signature

The functional enrichment analysis of miRNAs in the signature was applied by Gene Ontology (GO) annotation and Kyoto Encyclopedia of Genes and Genomes (KEGG) signaling pathway in miRPath v.3 (Vlachos et al., 2015).

### miRNA–Gene Interaction Network

We screened transcriptional target genes of miRNAs in our signature by the miRWalk database^2^ (Dweep and Gretz, 2015). Then, miRNA–gene interaction network with interacting pairs was visualized by Cytoscape (version 3.7.1) software^3^ (Shannon et al., 2003).

### Gene Expression Omnibus Data Validation

We then tested whether miRNAs in the signature were expressed differentially in another independent GEO database. GSE75968 consisted of 12 tumor samples and 12 paired normal tissues from patients with EEC from the GPL19117 platform. Probes were converted to the gene symbols based on a manufacturer-provided annotation file, and duplicated probes for the same gene were removed by determining the median expression value of all of its corresponding probes.

### Survival Analysis

To determine the association of specific miRNAs with survival, Kaplan–Meier survival analysis was performed using TCGA-UCEC database. Log-rank test was utilized for comparison of survival curves between “high” and “low” expression group. All statistical analyses were conducted using SPSS Version 23.0 software or R statistical software version 3.6.0. Two-tailed tests and p values < 0.05 for significance were used.

## Results

### Candidate miRNAs Screened by the Three Methods

#### Differential Expression Analysis

**FIGURE 2:**
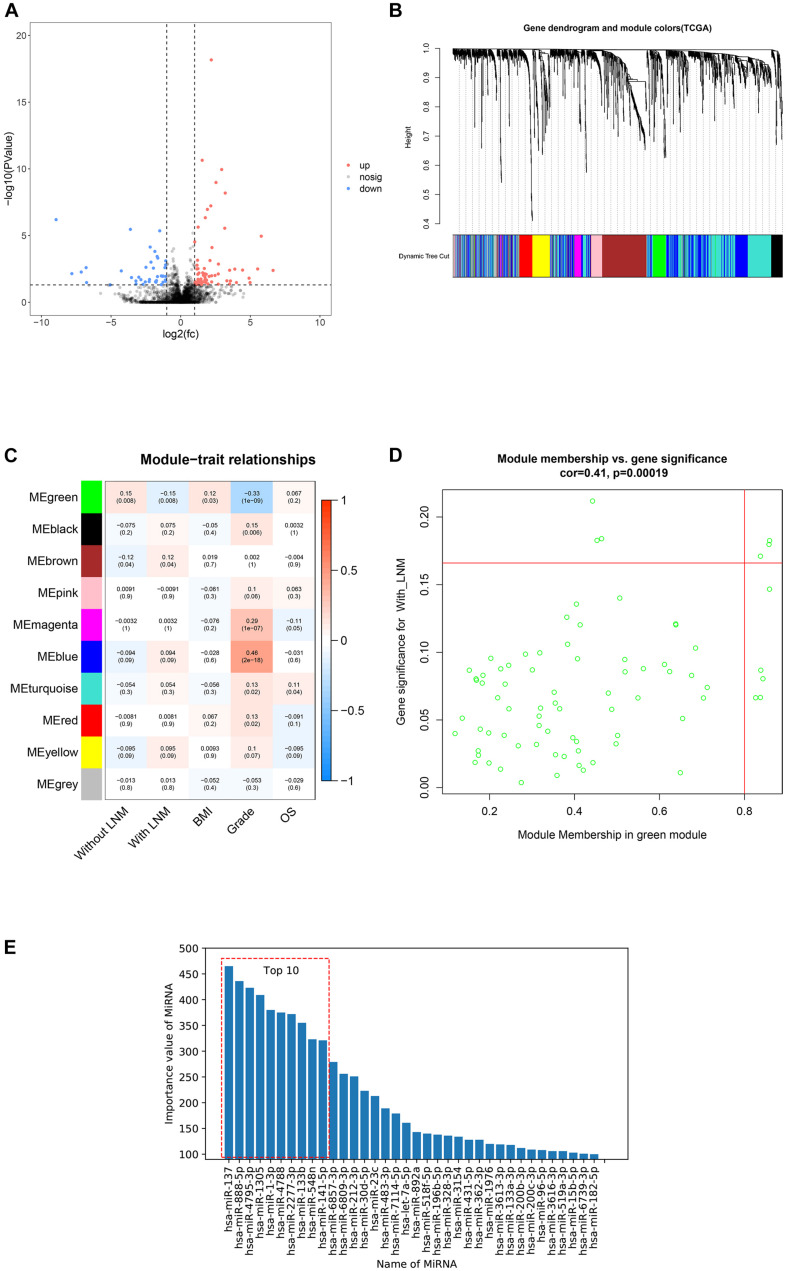
Candidate miRNAs screening by three methods. This figure describes the screening of candidate miRNAs by three methods. **(A)** Volcano plot of differentially expressed miRNAs between patients with lymph node metastasis (LNM) and without LNM. Red dots represented upregulated miRNAs, blue dots represented downregulated miRNAs, and black dots represented no significance. **(B)** Dendrogram of genes clustered based on dissimilarity measure. The upper panel showed the gene dendrogram, and the lower panel showed 10 gene modules displayed in different colors. **(C)** Heat map of the correlation between modules and clinical features. The number above each cell represented the correlation between the module and the feature, and the number below each cell represented the corresponding p value. Red represented a positive correlation, and blue represented a negative correlation. **(D)** Scatter plot showed the correlation between gene significance (GS) and module membership (MM) in the green modules. **(E)** The importance value of miRNAs in Gradient Boosting Decision Trees (GBDTs) by decision tree.

After filtering out excluded cases, 324 patients were selected for analyses. Here, 113 miRNAs were differently expressed between patients with and without LNM. Among them, 73 miRNAs were upregulated and 40 miRNAs were downregulated in patients with LNM (Figure 2A). Ten miRNAs with the most significant discrepancy were selected to construct the predictive signature.

#### Construction of Co-expression Network

To build a scale-free network, a soft threshold value (β) was tried from 1 to 20 following the WGCNA protocol. With β = 4, the degree of independence reached 0.9 and the mean connectivity dropped to zero, indicating that the network met the requirements for scale-free distribution. Gene modules close to each other were visualized by the dynamic tree cut method (Figure 2B). Finally, 10 modules were obtained, and only modules significantly correlated with certain clinical features were selected (Figure 2C). There was a significant negative correlation between the green module and LNM. Besides, correlation analysis showed that gene significance (GS) and module membership (MM) of the green modules were significantly correlated (cor = 0.41), suggesting that miRNAs in the green module may be related to LNM progression. Among miRNAs in the green module, hsa-miR-34b-3p, hsa-miR-34c-5p, hsa-miR-34c-3p, hsa-miR-449c-5p, hsa-miR-449b-5p, hsa-miR-34b-5p, hsa-miR-449a, hsa-miR-449b-3p, hsa-miR-10a-5p, hsa-miR-135a-3p, and hsa-miR-10a-3p were selected for building the predictive signature due to their high GS and MM (Figure 2D).

#### Decision Tree Analysis

The GBDT construction process was repeated 1,000 times with random super parameters. To ensure that the GBDT was not overfitted or underfitted, among the 1,000 models, only 147 GBDTs that met the criterion were selected. Then, we summed up the importance value of features in the aforementioned GBDTs for feature ranking and screened the top 10 as potential target miRNAs (Figure 2E).

Together with the three methods, a total of 31 miRNAs were screened for signature construction.

### Construction and Validation of the miRNA Signature

A total of 324 patients with an average age of 62.81 years were included in this study from TCGA-UCEC database, and 36 (11.1%) had LNM. They were randomly partitioned into a training cohort (*n* = 226) and a validation cohort (*n* = 98). As shown in Table 1, the demographics of the two cohorts were well balanced, including age, body mass index, the proportion of LNM, and G stage.

**TABLE 1:** Clinical Characteristic of the Training and Validation Cohorts.

	**Training cohort**	**Validation cohort**	**p value**
Number of patients	226	98	
LNM	25 (11.1%)	11 (11.2%)	0.966¶
Body mass index (kg/m^2^)	34.89 ± 9.19	33.37 ± 10.04	0.621*
Age (year)	62.35 ± 11.69	63.88 ± 10.67	0.491*
**Menopausal status**			
Premenopausal	17 (7.5%)	6 (6.1%)	0.095¶
Perimenopausal	11 (4.9%)	3 (3.1%)	
Postmenopausal	174 (77.0%)	86 (87.8%)	
Unknown	24 (10.6%)	3 (3.1%)	
**G stage**			
G1	54 (23.9%)	28 (28.6%)	0.612¶
G2	66 (29.2%)	29 (29.5%)	
G3	106 (46.9%)	41 (41.8%)	
**Surgical approach**			
Open	139 (61.5%)	56 (57.1%)	0.661¶
Minimally invasive	81 (35.8%)	38 (38.8%)	
Unknown	6 (2.7%)	4 (4.1%)	

Data are expressed as mean ± SD or number (percentage).

*The *p* value was calculated by the *t* test.

*¶The *p* value was calculated by the χ^2^ test.*

*LNM, lymph node metastasis.*

#### Construction of the miRNA Signature

Thirty-one screened miRNAs were entered into the logistic regression program in the training cohort. After removing 16 miRNAs that contributed little to the model, the final miRNA signature was defined. The 15 selected miRNAs were hsa-miR-449c-5p, hsa-miR-34b-5p, hsa-miR-34b-3p, hsa-miR-449b-3p, hsa-miR-34c-5p, hsa-miR-135a-3p, hsa-miR-34c-3p, hsa-miR-483-3p, hsa-miR-875-3p, hsa-miR-612, hsa-miR-122-5p, hsa-miR-137, hsa-miR-4795-3p, hsa-miR-4788, and hsa-miR-548n. A risk score of LNM in EEC was calculated according to the logistic regression formula as follows (displayed as a coefficient multiplied by miRNA′, which was calculated by dividing the miRNA count by the standard deviation; the complete formula for risk score calculation was shown in Supplementary Table 1):

r⁢i⁢s⁢k⁢s⁢c⁢o⁢r⁢e

$$=-0.2796*\text{h}\,s\,a\,m\,i\,R\,449\,{\text{c5p}}^{\prime }-0.4063*\text{h}\,s\,a\,m\,i\,R\,34\,{\text{b5p}}^{\prime }$$

$$-0.5534*\text{h}\,s\,a\,m\,i\,R\,34\,{\text{b3p}}^{\prime }+0.7191*\text{h}\,s\,a\,m\,i\,R\,449\,{\text{b3p}}^{\prime }$$

$$-0.4134*\text{h}\,s\,a\,m\,i\,R\,34\,{\text{c5p}}^{\prime }-1.4148*\text{h}\,s\,a\,m\,i\,R\,135\,{\text{a3p}}^{\prime }$$

$$+0.2727*\text{h}\,s\,a\,m\,i\,R\,4833\,{\text{p}}^{\prime }-0.3207*\text{h}\,s\,a\,m\,i\,R\,34\,{\text{c3p}}^{\prime }$$

$$+0.5091*\text{h}\,s\,a\,m\,i\,R\,8753\,{\text{p}}^{\prime }+0.3033*\text{h}\,s\,a\,m\,i\,R\,612^{\prime }$$

$$-0.6916*\text{h}\,s\,a\,m\,i\,R\,1225\,{\text{p}}^{\prime }+0.4155*\text{h}\,s\,a\,m\,i\,R\,137^{\prime }$$

$$+0.6475*\text{h}\,s\,a\,m\,i\,R\,47953\,{\text{p}}^{\prime }-0.2738*\text{h}\,s\,a\,m\,i\,R\,4788^{\prime }$$

$$+0.2404*\text{h}\,s\,a\,m\,i\,R\,548\,{\text{n}}^{\prime }-7.8298$$

#### The Prediction Confidence of the miRNA Signature

**FIGURE 3:**
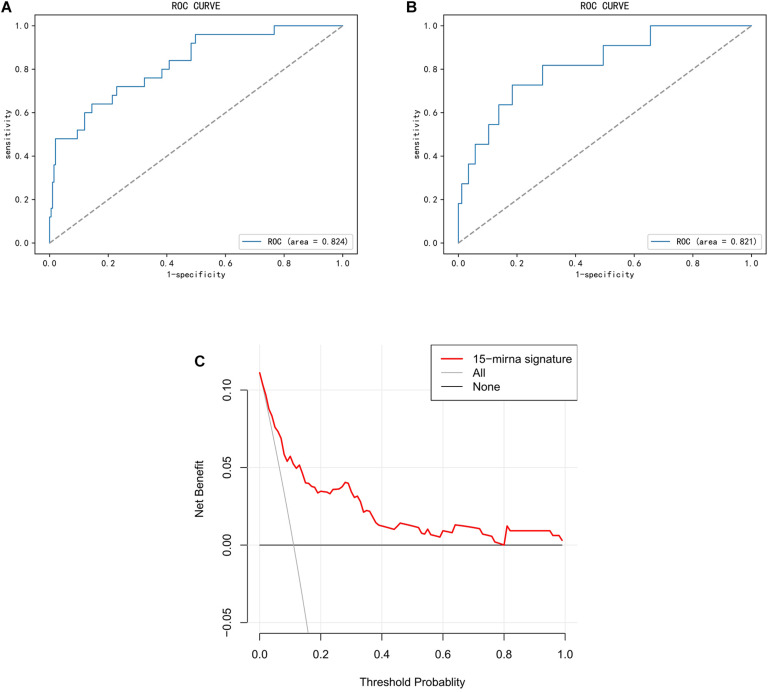
Validation of miRNA signature in predicting lymph node metastasis (LNM) in endometrial endometrioid carcinoma (EEC). This figure describes the performance of miRNA signature in predicting LNM in EEC. **(A,B)** Receiver operating characteristic curve (ROC) of the miRNA signature in the training cohort and the validation cohort. **(C)** Decision curve analysis (DCA) for the miRNA signature. The Y-axis represented net benefit. The X-axis represented threshold probability. The threshold probability was where the expected benefit of treatment is equal to the expected benefit of avoiding treatment. The red line represented the miRNA signature. The blue line represented the hypothesis that all patients had LNM. The black line represented the hypothesis that no patient had LNM.

The prediction confidence of the 15-miRNA signature was validated in the training and validation cohorts, with AUC of 0.824 (95% CI, 0.739–0.912) and 0.821 (95% CI, 0.691–0.925), respectively (Figures 3A,B). The result of DCA showed that the miRNA signature would be more clinically beneficial than the strategy “treat all” or “treat none” for predicting LNM if the threshold probability of a patient was between 0.1 and 0.8 (Figure 3C). Therefore, the results of ROC and DCA both proved that the miRNA signature had good predicted validation.

### The Gene Ontology Annotation and Kyoto Encyclopedia of Genes and Genomes Analysis

**FIGURE 4:**
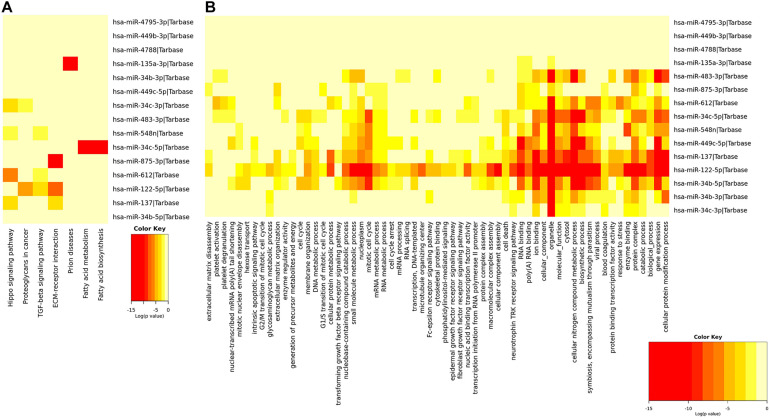
Functional enrichment analysis of miRNAs in the signature. This figure describes the functional enrichment analysis of miRNAs in the signature. **(A,B)** Gene Ontology (GO) analysis and Kyoto Encyclopedia of Genes and Genomes (KEGG) biological process analysis.

The functional enrichment analysis of miRNAs in the signature applied by GO annotation and KEGG signaling pathway was displayed in Figure 4. The result of GO annotation showed that miRNAs in the signature played roles in the mitotic cell cycle, cellular protein modification process, molecular function, and so on, some of which may make a contribution to the metastasis of EEC. The KEGG analysis suggested seven pathways were significantly enriched, including extracellular matrix (ECM)–receptor interaction, proteoglycans in cancer, transforming growth factor (TGF)-beta signaling pathway, and fatty acid metabolism.

### The Construction of miRNAs and mRNA Regulatory Network

**FIGURE 5:**
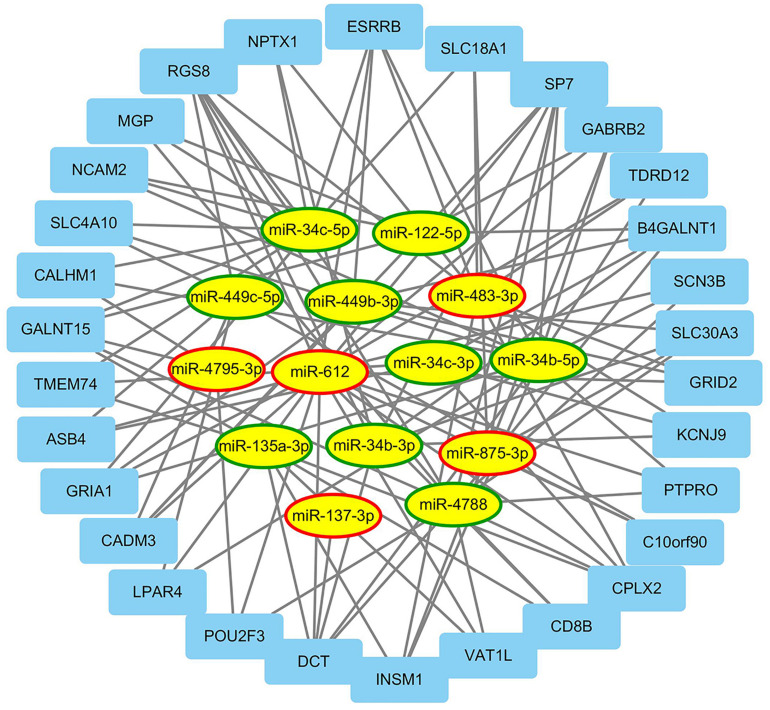
The regulatory network between miRNAs and differentially expressed mRNAs. This figure describes the regulatory network between miRNAs and differentially expressed mRNAs associated with lymph node metastasis (LNM) in endometrial endometrioid carcinoma (EEC). The ellipses and rectangles represented the miRNAs and mRNAs, respectively. The red and green rings indicated relatively upregulated and downregulated expression in EEC patients with LNM, respectively.

From TCGA database, a total of 188 mRNAs were differentially expressed between EEC patients with LNM and those without LNM (|fold change| > 2, FDR < 0.05). Using the miRWalk database, mRNAs targeted by miRNAs in our signature were identified, and 30 of the most related mRNAs were selected to construct a miRNA–mRNA regulatory network by Cytoscape 3.7. As shown in Figure 5, there were 114 interactions in this network. Among them, hsa-miR-135a-3p, hsa-miR-4788, and hsa-miR-122-5p regulated the most target mRNAs; meanwhile RGS8, DCT, and SP7 were regulated by most miRNAs.

### Gene Expression Omnibus Data Validation and Survival Analysis

**FIGURE 6:**
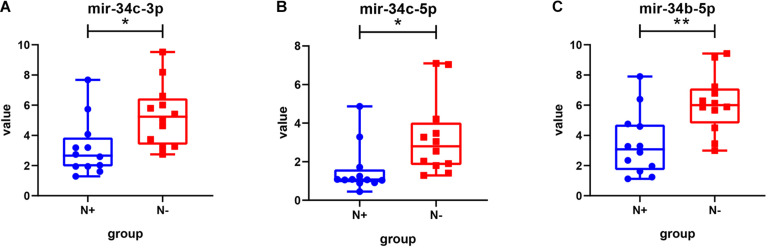
The expression value of miRNAs in different groups in the GSE75968 database. This figure describes the expression value of miR-34c-3p **(A)**, miR-34c-5p **(B)**, and miR-34b-5p **(C)** in lymph node metastasis-positive and lymph node metastasis-negative groups in the GSE75968 database. N+, lymph node metastasis positive; N-, lymph node metastasis negative. Data were shown as mean ± SD. Individual data points were shown. *p < 0.05, **p < 0.01.

**FIGURE 7:**
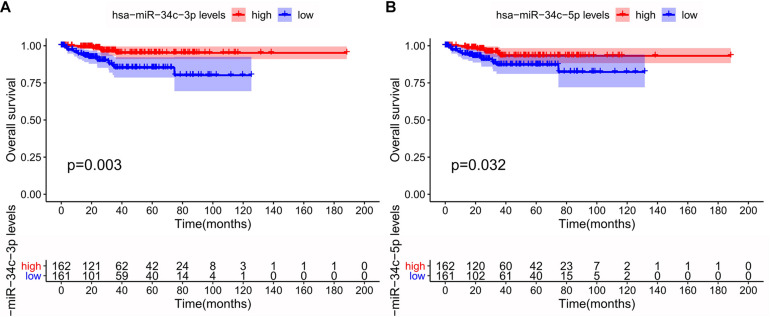
Survival analysis of the miR-34c-3p **(A)** and miR-34c-5p **(B)** in The Cancer Genome Atlas (TCGA) database. This figure describes the survival analysis (Kaplan–Meier plot) of the miR-34c-3p and miR-34c-5p in endometrial endometrioid carcinoma (EEC) in TCGA database. The red line and blue line represented groups with higher expression and lower expression of specific miRNA, respectively.

Among the 15 miRNAs, miR-34c-3p, miR-34c-5p, and miR-34b-5p were expressed differentially in GSE75968. The expression values of miR-34c-3p, miR-34c-5p, and miR-34b-5p in LNM-positive patients were significantly lower than those in LNM-negative patients (3.163 vs. 5.343, 1.557 vs. 3.259, 3.445 vs. 6.113, respectively), inferring that the miR-34 cluster may be key miRNA related to LNM progress (Figure 6). We then applied Kaplan–Meier survival analysis with miRNAs in our signature using TCGA-UCEC database. During the follow-up period, among 324 EEC patients, 30 died (9.26%) and one was lost to follow-up (0.31%). The 5-year overall survival rate was 88.9%. As shown in Figure 7, miR-34c-3p and miR-34c-5p were statistically significant in survival. Higher expression of miR-34c-3p and miR-34c-5p was associated with higher survival time. Thus, miR-34c was related to prognosis, and further research ought to be completed about the molecular mechanism of miR-34c in EEC.

## Discussion

Endometrial cancer is a major gynecological malignancy worldwide, with a cumulative risk of 1% by the age of 75 years, while the death risk is 0.2% (Morice et al., 2016; Van Nyen et al., 2018). LNM is a critical prognosis-related risk factor for EEC, and the status of lymph nodes is an essential consideration when making clinical decisions. Since lymphadenectomy is not applied as routine therapy in EEC, new ways for determining lymph node status need to be explored. Sentinel lymph node (SLN) mapping can be an alternative—thanks to its increased detection rate compared with lymphadenectomy (Ballester et al., 2011; Rossi et al., 2017). However, reliable SLN mapping requires surgeons and institutions to equip relevant expertise and skills. Also, SLN mapping is performed during surgery. Consequently, finding preoperative ways that can accurately identify LNM would have great clinical value. Similar to most tumors, the occurrence, development, and metastasis of EEC also involve complex molecular mechanisms (Stampoliou et al., 2016). Recently, research using dysregulated miRNAs as powerful tools to characterize environments of tumor and identify novel oncogenic pathway is emerging (Rupaimoole et al., 2016). Furthermore, there is a view that miRNA dysregulation patterns and signatures work better than mRNA in terms of identifying tumor origins due to their stability, robust expression, and lack of transcript variants (Chan et al., 2011). Thus, miRNAs may be reliable molecular biomarkers to predict LNM and help in the diagnosis and treatment of EEC.

For the first time, we developed a miRNA signature to predict LNM in patients with EEC using TCGA-UCEC cohort. Innovatively, we used three different methods to screen candidate miRNAs. Identifying differently expressed genes or miRNAs by fold change between two groups is the most common way to find out the hub biomolecules in present bioinformatics research. However, a disadvantage of using fold change is that it is biased and may misclassify differentially expressed genes with large differences but small ratios, leading to poor identification of changes at high expression levels (Mariani et al., 2003). Recently, WGCNA analysis is widely used to construct the modules of co-expression genes that relate to prognosis or other clinical outcomes. For instance, researchers found that Prostaglandin D2 Synthase (PTGDS) predicted poor survival, while ANO1 might be a potential marker for good prognosis in endometrial cancer by WGCNA (Wang et al., 2019; Zou et al., 2020). Besides, an increasing number of research apply the machine learning into the biomedical field. Decision tree algorithm is used to detect the important features in classification in the machine learning field and is also applicable for diagnosis and classification of diseases. By using the three aforementioned methods, 31 miRNAs were screened as candidate target miRNAs for signature construction. It should be noted that the screened miRNAs from each method were scarcely overlapped, indicating that the data were analyzed in discrepant statistical ways, which would make better use of specific data and lead to more discoveries.

We constructed the final 15-miRNA signature to predict the LNM of EEC by logistic regression, and a risk score of LNM was calculated. The AUC values were 0.824 and 0.821 in the training and validation cohorts, respectively. Thus, our miRNA signature has potential for LMN prediction and may provide biological insights in EEC. The result of DCA suggested that the miRNA signature had clinical value. The signature would be more beneficial than the strategy “treat all” or “treat none” in most cases, with a threshold probability range from 0.1 to 0.8. Subsequently, functional enrichment analyses were performed to define biological process, molecular function, and signaling pathways. Determination of these pathways could serve as potential therapeutic targets for treatments in EEC and help in future clinical use. Meanwhile, a miRNA–mRNA interaction network was visualized by Cytoscape. Identifying the interactions between miRNAs in our signature and mRNA did good on our understanding of the regulation of target miRNAs in EEC.

To validate whether miRNAs in the signature were expressed differentially in another independent database, we tested the expression of our miRNA between LNM-positive and LNM-negative groups in GSE75968. The same as what we found in TCGA-UCEC, the expression of miR-34c-3p, miR-34c-5p, and miR-34b-5p was significantly lower in the LNM-positive group. Moreover, miR-34c-3p and miR-34c-5p were statistically significant in survival. Higher expression of miR-34c-3p and miR-34c-5p was associated with longer survival time, indicating that miR-34c may be a key miRNA related to LNM progress and survival.

miRNAs in our signature were known to function in oncogenesis or had been reported to have prognostic value in cancers, especially in endometrial cancer. In our signature, hsa-miR-34c-5p, hsa-miR-34c-3p, hsa-miR-135a-3p, hsa-miR-449b-3p, hsa-miR-34b-5p, hsa-miR-34b-3p, hsa-miR-122-5p, hsa-miR-449c-5p, and hsa-miR-4788 were downregulated in patients with LNM in EEC. It was reported that overexpression of miR-34c-5p significantly inhibited cell proliferation, colony formation, migration, and invasion and induced cell cycle arrest and apoptosis by targeting E2F3 in HEC-1-B cells (Li et al., 2015). Liu et al. (2020) also found that miR-34a/c induced caprine endometrial epithelial cell apoptosis by regulating circ-8073/CEP55 *via* the RAS/RAF/MEK/ERK and PI3K/AKT/mTOR pathways. Simultaneously, miR-34 may have regulatory effects on epithelial–mesenchymal transition (EMT) of cancers by targeting SNAIL (Zhang et al., 2019). Studies have concluded that miR-34b might act as a tumor suppressor in endometrial serous adenocarcinoma, estrogen-dependent breast cancer, and lung cancer (Lee et al., 2011; Hiroki et al., 2012). The impact of miR-135 on endometrial cancer was contradictory in the literature. Wang J. et al. (2020) revealed that miR-135a promoted proliferation, migration, and invasion and induced chemoresistance of endometrial cancer cells, but (Mirabutalebi et al., 2018) found miR-135a acted as a tumor suppressor by targeting ASPH in endometrial cancer (Chen et al., 2019; Wang J. et al., 2020). A positive correlation was also observed between the expression of miR-135a and endometriosis lesions, which is a disease also referring migration of the endometrium (Mirabutalebi et al., 2018; Petracco et al., 2019). The expression of miR-449b was markedly reduced in type II endometrial cancer tissues, and its reduction was associated with endometriosis lesions *via* endometrial stromal cell proliferation and angiogenesis (Braza-Boils et al., 2014; Ye et al., 2014; Liu et al., 2018). Similarly, literature revealed that miR-449 suppressed endometrial cancer invasion and metastasis by targeting N-MYC downstream regulated gene 1 (NDRG1) (Wu et al., 2019).

On the other hand, hsa-miR-483-3p, hsa-miR-548n, hsa-miR-137, hsa-miR-612, hsa-miR-4795-3p, and hsa-miR-875-3p were upregulated in patients with LNM in EEC. miR-483 has not been reported to be associated with endometrial cancer. Nevertheless, miR-483-5p was significantly downregulated in patients with endometriosis (Laudanski et al., 2013). Zhu et al. (2020) reported that the unavailability of miR-548 suppressed the progression of colorectal cancer by regulating the miR-548n/TP53INP1 signaling pathway. Moreover, miR-548 downregulated the host immune response *via* direct targeting of IFN-λ1 and thereby might provide a better microenvironment for tumor progression (Li et al., 2013). The expression of miR-137 was higher in patients with LMM in TCGA-UCEC; however, others reported that miR-137 was a tumor suppressor in endometrial cancer and was repressed by DNA hypermethylation (Banno et al., 2014; Zhang W. et al., 2018). Zhang L. et al. (2018) found that miR-612 might compete with lncRNA H19 to regulate the expression of target gene HOXA10, which is related to cancer cell proliferation in endometrial carcinoma. Similarly, miR-612 was associated with esophageal squamous cell carcinoma development and metastasis, mediated through TP53 (Zhou et al., 2017).

Additionally, some mRNAs targeted by our identified miRNAs were reported to engage in tumorigenesis and progression. Zhang et al. (2009) found that Zinc finger transcription factor INSM1 interrupted cyclin D1 and CDK4 binding and induced cell cycle arrest. Besides, adjacent single-nucleotide polymorphisms (SNPs) to gene B4GALT1 could be associated with cervical cancer development (Danolic et al., 2020). Research revealed both SLC30A3 and GABRB2 had diagnostic and prognostic values for colon adenocarcinoma (Yan et al., 2020; Yin et al., 2020). As for Regulator of G Protein Signaling Like 1 (RGSL), its novel mutations were related to the pathophysiology of breast cancer (Wiechec et al., 2011). Cell Adhesion Molecule 3 (CADM3) engaged in retinoblastoma cell proliferation, migration, and invasion targeted by miR-140-5p (Miao et al., 2018).

Although miRNAs in our signature were reported to be closely related to occurrences and progression of tumors, the relationships between some miRNAs and EEC were uncertain. Furthermore, there is few research referring to the target gene regulated by those miRNAs and their interactions. Thus, investigations are warranted to look into these miRNAs and genes.

The current study has several limitations. The proportion of patients with LNM was low in TCGA database. Besides, both our training and validation cohorts were obtained from TCGA database. Thus, more EEC samples are needed for further validation of the constructed signature before application. One other limitation is that the mechanisms of most identified miRNAs of EEC were unclear, so downstream experimental studies on these miRNAs need to be completed in the future.

## Conclusion

In conclusion, we constructed a miRNA signature that worked as a noninvasive method to detect LNM in EEC and achieved a high prediction accuracy. In addition, miR-34c cluster may be key biomarkers referring LNM in endometrial cancer.

## Data Availability Statement

The datasets TCGA-UCEC and GSE-75968 for this study can be found in the TCGA (https://portal.gdc.cancer.gov/) and GEO (https://www.ncbi.nlm.nih.gov/gds).

## Author Contributions

JX designed the study. KF, YL, JS, WC, WW, and XY prepared material and collected and analyzed the data. KF wrote the first draft of the manuscript. All authors read and approved the final manuscript.

## Conflict of Interest

The authors declare that the research was conducted in the absence of any commercial or financial relationships that could be construed as a potential conflict of interest.

## Footnotes

1 https://portal.gdc.cancer.gov/

2 http://www.umm.uni-heidelberg.de/apps/zmf/miRwalk/

3 http://cytoscape.org/
