# Supplementary material for: Identification of a MicroRNA Signature Associated With Lymph Node Metastasis in Endometrial Endometrioid Cancer
Source: Front Genet. 2021 Apr 15;12:650102. doi: 10.3389/fgene.2021.650102 (PMC8082502; doi:10.3389/fgene.2021.650102)
Supplement: Supplementary file 1 [file Table_1.DOCX]

Supplementary Material

**Supplementary Table 1 The Complete Formula for Risk Score Calculation**

| The Complete Formula | $\boldsymbol{risk score=-0.00212363*hsamiR}\boldsymbol{449}\boldsymbol{c}\boldsymbol{5}\boldsymbol{p-0.00150919*hsamiR}\boldsymbol{34}\boldsymbol{b}\boldsymbol{5}\boldsymbol{p-0.00266496*hsamiR}\boldsymbol{34}\boldsymbol{b}\boldsymbol{3}\boldsymbol{p+0.00777614*hsamiR}\boldsymbol{449}\boldsymbol{b}\boldsymbol{3}\boldsymbol{p-0.000341978*hsamiR}\boldsymbol{34}\boldsymbol{c}\boldsymbol{5}\boldsymbol{p-0.0272633*hsamiR}\boldsymbol{135}\boldsymbol{a}\boldsymbol{3}\boldsymbol{p+0.000418594*hsamiR}\boldsymbol{4833}\boldsymbol{p-0.000298763*hsamiR}\boldsymbol{34}\boldsymbol{c}\boldsymbol{3}\boldsymbol{p+0.0571054*hsamiR}\boldsymbol{8753}\boldsymbol{p+0.104274*hsamiR}\boldsymbol{612-0.0012512*hsamiR}\boldsymbol{1225}\boldsymbol{p+0.059925*hsamiR}\boldsymbol{137+1.15285*hsamiR}\boldsymbol{47953}\boldsymbol{p-0.018241*hsamiR}\boldsymbol{4788+0.366187*hsamiR}\boldsymbol{548}\boldsymbol{n-7.82979941}$ |
| --- | --- |

Note that the counts of expression of miRNAs before normalization were used in this formula which can be popularized.
